# The novel nitric oxide donor PDNO attenuates ovine ischemia-reperfusion induced renal failure

**DOI:** 10.1186/s40635-017-0143-4

**Published:** 2017-06-09

**Authors:** Kristofer F. Nilsson, John Sandin, Lars E. Gustafsson, Robert Frithiof

**Affiliations:** 1 Department of Cardiothoracic and Vascular Surgery, Faculty of Medicine and Health, Örebro University, Örebro, Sweden; 2 Department of Physiology & Pharmacology, Karolinska Institutet, Stockholm, Sweden; 3 Department of Surgical Sciences, Section of Anesthesia and Intensive Care, Uppsala University, Uppsala, Sweden

**Keywords:** Kidney, Microcirculation, Sheep, Renal oxygen consumption, Nitrites, Acute kidney injury, AKI

## Abstract

**Background:**

Renal ischemia-reperfusion injury is a common cause of acute kidney injury in intensive care and surgery. Recently, novel organic mononitrites of 1,2-propanediol (PDNO) were synthesized and shown to rapidly and controllably deploy nitric oxide in the circulation when administered intravenously. We hypothesized that intravenous infusion of PDNO during renal ischemia reperfusion would improve post-ischemic renal function and microcirculation.

**Methods:**

Sixteen sheep were anesthetized, mechanically ventilated, and surgically instrumented. The left renal artery was clamped for 90 min, and the effects of ischemia were studied for a total of 8 h. Fifteen minutes prior to the release of the clamp, intravenous infusions of PDNO (*n* = 8) or vehicle (1,2 propanediol + inorganic nitrite, *n* = 8) were initiated (180 nmol/kg/min for 30 min, thereafter 60 nmol/kg/min for the remainder of the experiment).

**Results:**

Renal artery blood flow, cortical and medullary perfusion, and diuresis and creatinine clearance decreased in the left kidney post ischemia. However, in the sheep treated with PDNO, diuresis and creatinine clearance in the left kidney were significantly higher post ischemia compared to vehicle-treated animals (1.7 ± 0.5 vs 0.7 ± 0.3 ml/kg/h, *p* = 0.04 and 7.5 ± 2.1 vs 1.7 ± 0.6 ml/min, *p* = 0.02, respectively). Left renal medullary perfusion and oxygen uptake were higher in the PDNO group (73 ± 9 vs 37 ± 5% of baseline, *p* = 0.004 and 2.6 ± 0.4 vs 1.6 ± 0.3 ml/min, *p* = 0.02, respectively). PDNO significantly increased renal oxygen consumption and reduced the oxygen utilization for sodium reabsorption (*p* = 0.03 for both). Mean arterial blood pressure was significantly reduced by PDNO (83 ± 3 vs 94 ± 3 mmHg, *p* = 0.02) but was still within normal limits. Total renal blood flow was not affected, and there were no signs of increased blood methemoglobin concentrations or tachyphylaxis.

**Conclusions:**

The novel nitric oxide donor PDNO improved renal function after ischemia. PDNO also prevented the persistent reduction in medullary perfusion during reperfusion and improved renal oxygen utilization without severe side effects.

## Background

Acute kidney injury (AKI) entails an increased risk of mortality and later development of chronic kidney disease in both critically ill and surgical patients [1, 2]. Originally, reduced blood flow has been overestimated as a cause of AKI, especially in sepsis [3]. Still warm ischemia is most likely a central component in the pathogenesis of AKI during prolonged hypotension, hypovolemia, and when blood flow is disrupted during cardiovascular events or vascular surgery [4]. Inadequate perfusion causes a mismatch in oxygen supply/demand in the renal tissue and scants removal of metabolic waste products, which result in injury and necrosis of renal tubular cells [5]. Ischemia also causes lingering vascular effects, including endothelial dysfunction, increased vascular tone, and a dysfunctional microcirculation, which further diminish renal function even if blood flow returns [6–8]. Several of these effects have been related to the release of free oxygen radicals [9].

Nitric oxide (NO) has been implicated on several stages in the pathogenesis of ischemic AKI. One hypothesis suggests that in renal ischemia-reperfusion injury (IRI), there is a reduction in endothelial nitric oxide synthase (NOS) but an increase in inducible NOS. This combination contributes to inflammation and vasoconstriction during reperfusion [10]. NO is a potent vasodilator as well as a free oxygen scavenger, which in theory would be beneficial post ischemia. However, treatment with exogenous NO in experimental renal IRI has resulted in contrasting results including beneficial, detrimental, or no effects on renal function [11–15]. This could be model specific and related to species but also coupled to the NO donor used.

Recently, a mixture of novel organic mononitrites of 1,2-propanediol was synthesized, chemically characterized, and shown to be an efficacious vasodilator treatment in experimental pulmonary hypertension [16]. The advantages of this composition of organic nitrites include very rapid deployment of NO in the tissue, easily controllable intravenous use, and minor development of methemoglobin. The fast donation of NO is theoretically important during initial reperfusion since the treatment may reach and have effect in the microcirculation before vascular dysfunction occurs. Furthermore, it contributes to a short plasma half-life of the compound, which in turn enables rapid control of the physiological response by adjusting the intravenous infusion rate of PDNO.

Based on these properties of PDNO and the pathophysiology of renal IRI, we hypothesized that intravenous infusion of PDNO would improve renal function and microcirculation after renal ischemia, without causing methemoglobinemia or severe systemic hypotension.

## Methods

The experimental protocol was approved in advance by the regional animal ethics committee in Stockholm, Sweden and adheres to the Laboratory Animal Care formulated by the National Society for Medical Research and the US National Academy of Sciences.

### Animals and surgical preparation

Adult cross-bred ewes (*n* = 16) weighing 61 ± 2 kg were used. They were housed individually in cages with free access to water. Twice a day, they were fed hay and 75 g commercial pellets with the addition of 6 g NaCl. All experiments started between 08.30 and 09.30 h, approximately 1–2 h after the morning feed.

Surgery was performed aseptically and under general anesthesia as previously described [17]. Anesthesia was induced by an intravenous injection of sodium thiopental (15 mg/kg) in 0.9% saline. After endotracheal intubation, surgical anesthesia was maintained by isoflurane (2.1–2.3% end-tidal concentration) in an O_2_/air mixture (30%/70%) from a respirator, intravenous midazolam (5 mg in 50 ml isotonic NaCl, 0.05 mg/kg/h), and intravenous administration of fentanyl (50–100 μg). Tidal volumes were kept at 10 ml/kg, and the respiratory rate was adjusted to keep end-tidal CO_2_ between 5.1 and 5.9%. The left carotid artery was surgically dissected and cannulated for blood sampling and measurement of arterial blood pressure. The skin and underlying tissue layers were enclosed with sutures. The sheep were prepared with a pulmonary artery catheter for measurement of cardiac output (CO) and central venous catheters as previously described [18, 19]. A flank incision was made from the last rib to the iliac crest to get a retroperitoneal approach to the aorta, left renal vein, left renal artery, and left kidney. A ligature was placed around the left renal artery at its departure from the aorta, which was later used as a vascular occluder to induce renal ischemia. Thereafter, an ultrasonic flow probe (4SB Transonic System Inc, NY, USA) was placed around the left renal artery distal to the ligature. The left renal vein was cannulated close to vena cava inferior, and a catheter was advanced in the vessel until it was located 5 mm from the kidney. This catheter was later used for renal venous blood sampling. The left ureter was then localized, cannulated, and ligated distally for urine collection from the left kidney. Urine from the right kidney was collected from the urine bladder via a retention catheter. Finally, a laser Doppler probe (0.25 mm fiber separation, 780 nm wavelength; Perimed AB, Järfälla, Sweden) was sutured to the surface of the kidney for cortical measurements, and a needle laser Doppler probe (0.15 mm fiber separation, 780 nm wavelength) was inserted 10–12 mm into the kidney for medullary measurements.

### Hemodynamic recordings

Arterial blood pressure was measured via a pressure transducer (DPT-6003, PVB Medizin Technik, BMBH, Kirchseen, Germany). Signals from the thermodilution pulmonary artery catheter were fed into a Vigilance® Monitor (Baxter Healthcare Corporation, CA, USA) where the CO was calculated by three consecutive injections (10 ml) of ice-cooled isotonic saline. The flow probe was connected to a Transonic T208 two channel flowmeter, and left renal volume blood flow was recorded continuously. Continuous online data acquisition was achieved by using the MP150/Acknowledge 3.9.1 system (BIOPAC Systems; Goleta, CA, USA) with a sampling rate of 200 Hz. The two laser Doppler probes were calibrated according to the manufacturer’s instructions and connected to a Periflux 5001 base unit (780 nm wavelength, 15 kHz band width, 0.2-s time constant; Perimed AB).

### Experimental protocol

**Fig. 1:**
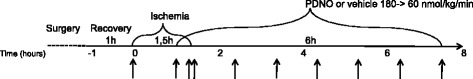
Schematic diagram illustrating the experimental protocol. After surgery, the sheep recovered for 60 min. Renal ischemia was caused by clamping of the left renal artery for 90 min. Fifteen minutes prior to the release of the clamp, intravenous infusions of either the organic mononitrites of 1,2-propanediol (PDNO) or vehicle (1,2-propanediol + inorganic nitrite) were commenced. The infusions continued for 6 h, during which renal function was monitored. The *arrows* indicate times for measurements and blood-/urine-sampling performed at baseline, prior to treatment, prior and directly following the release of the clamp, and continuously every hour after treatment was started

The experimental protocol is outlined in Fig. 1. After surgery, the sheep were allowed to recover for 60 min, during which urine was collected. Ischemia was then induced by complete clamping of the left renal artery via the surgically implanted ligature. The effect of the clamp was confirmed by a cease in measured left renal blood flow. Ischemia continued for 90 min before the clamp was released. The reperfusion phase was followed for an additional 6 h. Sheep were randomized to either receive an infusion of PDNO (17 mM PDNO, 11 mM inorganic nitrite, and 25% 1,2-propanediol [*v*/*v*] in saline, *n* = 8) or vehicle (28 mM inorganic nitrite and 25% 1,2-propanediol [*v*/*v*] in saline *n* = 8). The infusion was commenced 15 min prior to the release of the clamp at a rate of 180 nmol/kg/min of PDNO in the PDNO group. After 30 min, the infusion rate was reduced to 60 nmol/kg/min of PDNO in the PDNO-treated animals and was kept at this rate for the remainder of the experiment. The infusion of vehicle in the vehicle-treated animals was done at similar infusion rates in ml/kg/h as in the PDNO group. Thus, the vehicle-treated animals received an identical load of nitric oxide species in the form of inorganic nitrite that the PDNO group received in PDNO and inorganic nitrite as well as an identical load of 1,2-propanediol. The organic mononitrites of 1,2-propanediol was synthesized as previously described, and the inorganic nitrite content in the PDNO solution was a consequence of the method of synthesis [16]. Blood samples were taken from the left renal vein, carotid artery, and jugular vein at baseline, prior to treatment, prior and directly following the release of the clamp, and every hour after treatment was started. Urinary output was measured, and urine samples from both kidneys were collected at baseline, after 90 min and thereafter every second hour.

Fluid volume support was administered as intravenous glucose (50 mg/ml at 1 ml/kg/h) and Ringer’s acetate solution (7 ml/kg/h). The latter entails a substantial loading of fluids and electrolytes that may be considered protective during I/R. Both groups received the same fluid volume support in order not to influence the results of PDNO treatment.

### Blood, plasma, and urine analyses

The venous blood was portioned into pre chilled tubes with EDTA as anticoagulant and centrifuged at +4°C (3000 rpm). Plasma and urine aliquots were stored at −20 °C until assayed for creatinine according to the Jaffe method (Synchron LX, Beckman Instruments, Richmond, CA). Other portions of plasma and urine were taken for determination of sodium concentrations (IL 943 flame photometer; Instrumentation Labs, Italy). The carotid and renal venous blood samples were used for immediate arterial blood gas analyses performed on an ABL 700 (Radiometer, Copenhagen, Denmark).

### Calculations and statistical analysis

Cardiovascular parameters were averaged offline. Creatinine clearance was calculated as [(Urine flow × Urine creatinine concentration)/plasma creatinine concentration] and used as an estimate of GFR. Renal oxygen consumption (QO_2_) was calculated as [Renal blood flow × 1.34 × hemoglobin concentration × (arterial blood oxygen saturation − renal vein blood oxygen saturation)]. The oxygen consumed for sodium reabsorption was calculated as [renal oxygen uptake/(plasma sodium concentration × GFR − urinary sodium concentration × urine output)].

All statistical calculations were performed using Statistica 13 (Dell Inc., Tulsa, OK, USA), and the graphs were created with Sigma Plot 13 (SPSS Inc., Chicago, IL, USA). Data are expressed as mean ± standard error of the mean (SEM). The oxygen consumed for sodium reabsorption values and urine output were transformed to follow a normal distribution by taking the natural logarithm of the raw data. Changes in parameters over time were analyzed according to a two-way repeated measures ANOVA, followed by pairwise planned comparisons. The significance level was set at *p* ≤ 0.05.

## Results

All animals survived the experiment. CO and heart rate did not change significantly during the experiment (Table 1). Mean arterial blood pressure (MAP) was immediately slightly reduced by PDNO and remained significantly lower compared to vehicle (83 ± 3 vs 94 ± 3 mmHg, *p* = 0.02, after 6 h and 15 min of infusion, Table 1). There were no signs of tachyphylaxis with regards to the MAP response. In the vehicle-treated animals, methemoglobin levels were 3.4 ± 0.1% at baseline and 3.4 ± 0.2% at the end of the experiment. In the PDNO-treated sheep, the corresponding values were 3.3 ± 0.1% and 3.6 ± 0.1%, respectively.

**Table 1:** Hemodynamic parameters

Time (h)		0		1.25		1.5		1.6		2.5		3.5		4.5		5.5		6.5		7.5	
		Mean	SEM	Mean	SEM	Mean	SEM	Mean	SEM	Mean	SEM	Mean	SEM	Mean	SEM	Mean	SEM	Mean	SEM	Mean	SEM
Cardiac output (L/min)	Vehicle	8.4	0.6	7.7	0.4	7.7	0.5	7.9	0.6	7.6	1.3	9.0	0.4	8.6	0.3	8.5	0.2	8.3	0.3	7.8	0.3
	PDNO	7.2	0.3	6.5	0.6	6.6	0.5	6.5	0.4	8.1	0.7	8.5	0.5	8.3	0.3	8.2	0.4	8.3	0.5	7.7	0.4
Heart rate (BPM)	Vehicle	110	4	103	5	106	5	107	4	109	3	113	2	107	3	109	4	108	4	102	3
	PDNO	108	3	95	8	111	4	114	5	116	5	115	3	109	2	109	5	112	3	108	3
Mean arterial blood pressure (mmHg)	Vehicle	100	2	107	5	102	4	100	4	96	4	96	4	95	3	93	3	93	4	94	3
	PDNO	95	3	104	6	88	4	82	3	85	2	83	2	82	2	82	2	81	2	83*	3

Cardiovascular data from 16 anesthetized sheep subjected to left renal ischemia and reperfusion. Renal ischemia was caused by clamping of the left renal artery for 90 min. Fifteen minutes prior to the release of the clamp, intravenous infusions of either the organic mononitrites of 1,2-propanediol (PDNO, *n* = 8) or vehicle (propanediol + inorganic nitrite, *n* = 8) was commenced. The infusions continued for 6 h. Data are expressed as mean and SEM.

*Significant (*p* < 0.05) differences in response to PDNO compared to vehicle at the end of the infusion

### Renal function

**Fig. 2:**
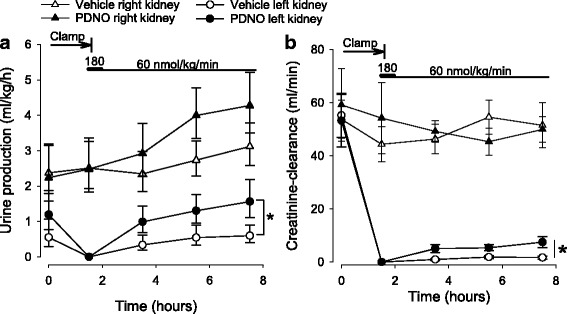
Urine output (**a**) and creatinine clearance (**b**) in 16 anesthetized sheep subjected to left renal ischemia and reperfusion. Renal ischemia was caused by clamping of the renal artery for 90 min. Fifteen minutes prior to the release of the clamp, intravenous infusions with either the organic mononitrites of 1,2-propanediol (PDNO, *n* = 8) or vehicle (1,2-propanediol + inorganic nitrite, *n* = 8) were commenced. The infusions continued for 6 h. Data are expressed as mean and SEM. Significant (*p* < 0.05) differences in response to PDNO compared to vehicle is indicated by an *asterisk*

Urine output and creatinine clearance were measured separately in each kidney. Both variables were reduced to zero in the left kidney in response to clamping of the left renal artery (Fig. 2a). There was a tendency to a slight but passing reduction in urine output also in the right kidney but neither urine output nor creatinine clearance changed significantly over time in the right kidney (Fig. 2a). Urine output from the left kidney increased post ischemia to a higher level in the sheep subjected to PDNO compared to vehicle (*p* = 0.04, Fig. 2a). The same effect was seen in the GFR estimation as the PDNO-treated sheep had a significantly higher creatinine clearance at the end of the experiment (*p* = 0.02, Fig. 2a). Plasma potassium increased from 3.5 ± 0.1 mmol/L at baseline to 4.0 ± 0.1 mmol/L 6 h post ischemia, with no intergroup differences.

### Renal hemodynamics

**Fig. 3:**
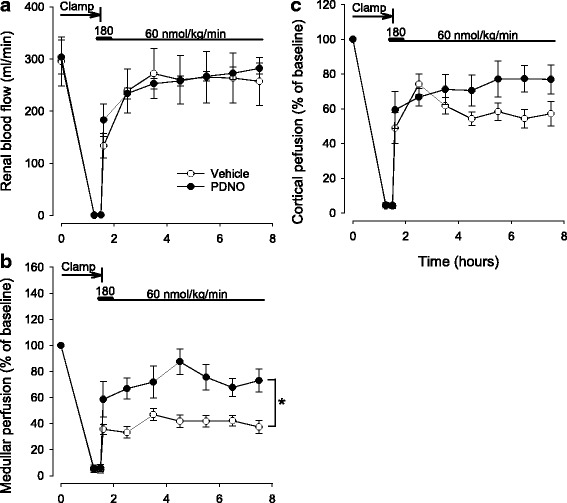
Total left renal blood flow (**a**) as well as left renal medullar (**b**) and cortical perfusion (**c**) as estimated by laser Doppler in 16 anesthetized sheep subjected to left renal ischemia and reperfusion. Renal ischemia was caused by clamping of the left renal artery for 90 min. Fifteen minutes prior to the release of the clamp intravenous infusions of either the organic mononitrites of 1,2-propanediol (PDNO, *n* = 8) or vehicle (1,2-propanediol + inorganic nitrite, *n* = 8) were commenced. The infusions continued for 6 h. Data are expressed as mean and SEM. Significant (*p* < 0.05) differences in response to PDNO compared to vehicle is indicated by an *asterisk*

Left renal blood flow was reduced to zero during clamping. After the release of the clamp, renal blood flow initially increased rapidly and had returned to near baseline levels at the end of the experiment (Fig. 3a). There were no significant effects of PDNO on total renal blood flow compared to vehicle. As expected, left renal perfusion ceased during the ischemia period but when renal blood flow returned, medullary perfusion increased to a higher level in the PDNO group compared to the vehicle group (*p* = 0.002, Fig. 3b). There was a similar tendency also in cortical microcirculatory perfusion, but this effect was not statistically significant (*p* = 0.08, Fig. 3c).

### Renal metabolism

**Fig. 4:**
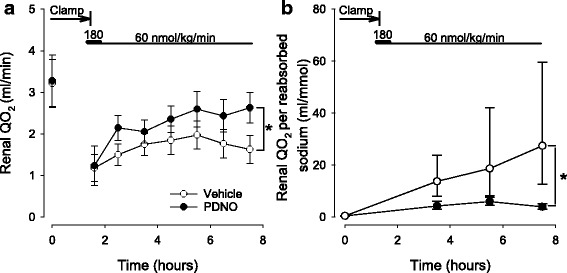
Renal oxygen consumption (QO_2_, **a**) and renal QO_2_ per reabsorbed sodium (**b**) in 16 sheep subjected to renal ischemia and reperfusion. Renal ischemia was caused by clamping of the renal artery for 90 min. Fifteen minutes prior to the release of the clamp intravenous treatment with either 1,2-propanediol nitric oxide (PDNO, *n* = 8) or vehicle (propanediol + inorganic nitrite, *n* = 8) was commenced. The treatment continued for 6 h. Data are expressed as mean and SEM. Significant (*p* < 0.05) differences in response to PDNO compared to vehicle is indicated by an *asterisk*

Left renal QO_2_ was similar in both groups at baseline. After the ischemic period, left renal QO_2_ was reduced in animals treated with PDNO and vehicle. However, although baseline levels were never reached, left renal QO_2_ slowly recovered during reperfusion. This effect was more pronounced in the PDNO group, resulting in a significantly higher left renal QO_2_ at the end of the experiment (*p* = 0.03, Fig. 4a). Renal tubular integrity can be estimated by calculating the amount of oxygen that is needed for the reabsorption of sodium. An increase in required oxygen indicates that sodium leaks back into the urine after reabsorption or that the active transport of sodium is damaged. In these experiments, the ratio between renal QO_2_ and reabsorbed sodium increased in response to ischemia and reperfusion indicating that the renal tubuli were damaged. However, PDNO attenuated this effect and after 6 h of reperfusion, the amount of oxygen required for sodium reabsorption was significantly lower in the PDNO-treated animals compared to vehicle (*p* = 0.03, Fig. 4b).

## Discussion

The most important finding in the current study was that the novel NO donor PDNO improved renal function, as estimated by urine output and creatinine clearance (Fig. 2), compared to a vehicle containing comparable amounts of inorganic nitrite. This was achieved without massive vasodilation and thus, although reduced, arterial blood pressure was maintained within limits not requiring vasopressor support. Furthermore, a 6 h infusion of PDNO did not cause methemoglobinemia.

Renal IRI is the result of direct ischemia causing acute tubular necrosis which subsequently leads to decreased glomerular filtration pressure and tubular obstruction [20]. In addition, reperfusion brings microcirculatory dysfunction and disturbed oxygen handling [21, 22]. All together, this gives rise to the decrease in GFR and urine output which is the clinical hallmarks of AKI [23]. The fact that PDNO was able to improve both these variables (Fig. 2) in a clinically relevant large animal model is important as it, for the first time, indicates that this new NO donor could be a future tool in the treatment of AKI induced by ischemia reperfusion.

Blood flow to the kidney is normally approximately 20% of CO and kept within relatively strict limits due to autoregulation [24]. The absolute majority of renal blood flow is directed to the renal cortex whereas the renal medulla only receives a fraction of the total renal blood flow. The high metabolic activity in combination with the relatively low blood supply makes the renal medulla susceptible to reductions in oxygen delivery [25]. In IRI, perfusion in the renal medulla has been shown to be persistently depressed [22, 26]. In the current experiments, total renal blood flow rapidly returned to near baseline values (Fig. 3a). However, in the vehicle group renal medullary perfusion only reached approximately 40% of baseline levels on a group level (Fig. 3b). Previous investigations indicate that this may be due to trapping of red blood cells, vasoconstriction, swelling of the endothelium and/or tubular obstruction [22, 26–28]. PDNO effectively improved medullary perfusion immediately after blood flow to the kidney returned (Fig. 3b). The rapid effect is in line with the ability of PDNO to swiftly deploy NO in the tissue, conceivably before some of the detrimental effects of reperfusion occur. NO acts as a vasodilator counteracting the ensuing vasoconstriction caused by endothelin-1, adenosine, and angiotensin II [29, 30]. It also scavanges free oxygen radicals which are believed to impair renal microcirculation in IRI [31]. In renal IRI, the vasorelaxing effect of acetylcholine is impaired [32] and NO production is depressed due to endothelial injury and reduced NOS activity [33–35]. Furthermore, it has been suggested that ROS produced during reperfusion react with and downregulate the bioavailability of NO causing medullary vasoconstriction [36]. Dysregulation of renal NO content thus likely contributes to vasoconstriction and hypoxia but could theoretically be counteracted by the exogenous NO donated by PDNO.

Renal oxygen handling is severely impaired in renal IRI [21, 37]. In the sheep subjected to IRI in this study, renal QO_2_ was significantly reduced post ischemia (Fig. 4a). Oxygen uptake in the kidney is high compared to other organs; only the heart has higher QO_2_ per gram tissue [38]. The most energy-consuming process is sodium reabsorption in the proximal tubule and the medullary thick ascending limb, and this transport is tightly coupled to GFR [39]. Thus, the reduced renal QO_2_ seen here may be due to major tubular cell death lowering oxygen requirements or to the reduced GFR, as estimated by creatinine clearance. An additional explanation is that the diminished renal QO_2_ post ischemia is related to reduced oxygen delivery caused by the impaired medullary perfusion (Fig. 3b). PDNO effectively improved renal QO_2_ (Fig. 4a), and based on the previous discussion, this is likely due to that NO prevented acute tubular necrosis, improved GFR, and/or renal perfusion.

At baseline, the sheep consumed approximately 0.5 ml oxygen per millimole of reabsorbed sodium which is slightly less compared to man [37]. After ischemia/reperfusion, this increased to an average of 27.4 ml oxygen per millimole of reabsorbed sodium in the vehicle group. A possible mechanism for this is that the renal tubular cells loose the integrity of the tight junctions due to ischemia and that NaK-ATPase redistributes to the apical membrane of tubular cells [40]. This in turn causes backward leakage of the reabsorbed sodium to the tubular lumen and faulty active transport of sodium from the intracellular domain to the urine [41]. If the function of the NaK-ATPase is intact, this will lead to increased oxygen-dependent transport of sodium without effective reabsorption. The PDNO-treated sheep displayed a significantly lower ratio between consumed oxygen and reabsorbed sodium, perhaps indicating less ischemic injury to tubular function.

NO is a key molecule in the control of renal oxygen supply, both as a vasodilator but also as regulator of oxidative metabolism [42]. An additional explanation for the increased oxygen utilization for sodium reabsorption that also is in line with the positive effect of NO deployment by PDNO is the decreased renal NO concentration previously discussed. Like oxygen, NO enters mitochondrial respiration and a decreased NO concentration augments oxygen consumption [43]. This is believed to play an important role in chronic kidney disease [44]. In instrumented conscious dogs, inhibition of NO production significantly increased the renal QO_2_/sodium reabsorption ratio [42]. Furthermore, recent data showed that the ROS-scavanger tempol prevented a fall in NO levels and improved tissue oxygen concentration after renal ischemia [45]. Taken together, this supports the idea that PDNO rapidly replaces pathologically decreased renal NO levels to improve oxygenation in the current experimental setup.

Methemoglobin reduces the oxygen carrying capacity of erythrocytes and impairs oxygen off-loading to the tissues [46]. Methemoglobinemia is a known side effect of both intravenous and inhalational NO treatments, most often after high doses have been administered [47, 48]. Our results indicate that PDNO can be infused intravenously at a rate sufficient for beneficial systemic effects without causing methemoglobinemia.

There are several limitations to this study that needs to be acknowledged. We aimed to use a model that mimics the clinical situation seen in renal IRI using fluid-resuscitated large animals. Still, results from animal models are not directly applicable to the human clinical scenario. What is most noteworthy is that many cases of IRI do not develop after a short period of total absence of blood flow but are instead the result of a longer period of insufficient perfusion, such as prolonged hypotension or hypovolemia. The current model perhaps mostly resembles suprarenal artery vascular surgery, although the reduction in creatinine clearance in the left kidney is more pronounced compared to what is usually seen clinically. Although the amelioration in creatinine clearance in the PDNO-treated animals was statistically significant, the improvement in absolute numbers was minor. This finding might suggest that PDNO is not sufficiently effective to be of clinical interest. To indicate a relevant effect on GFR, future experimental studies need to demonstrate a greater magnitude of protection by PDNO. It remains to be determined if this could be achieved by investigating the drug in a model with less pronounced decrease in creatinine clearance induced by a shorter period of complete renal ischemia or a subtotal reduction in renal blood flow. Furthermore, renal histology was not compared between groups. Although renal dysfunction is not always coupled to structural damage [49], this could have added additional mechanistic insight into the effects of PDNO. Another limitation is related to PDNO treatment. We used pre-treatment, which is not always clinically possible, and we did not compare PDNO with another classic NO donor but with inorganic nitrite (the vehicle). Inorganic nitrite was included in the placebo vehicle since the synthesis of PDNO also forms a certain amount of inorganic nitrite in the solution [16]. The vehicle-treated group received a solution with 28 mM of inorganic nitrite to achieve a similar load of nitric oxide species as the PDNO group (17 mM of PDNO and 11 mM of inorganic nitrite). Previously, inorganic nitrite administration has been suggested to reduce myocardial and hepatic IRI in several animal models [50]. In contrast, intravenously administered inorganic nitrite was shown to exert no effect on renal IRI whereas topically applied inorganic nitrite improved renal function in renal IRI [51, 52]. From the present experiments, it is not possible to estimate the effects of inorganic nitrite on renal IRI since the vehicle in the control group also included inorganic nitrite. However, based on previous investigations in the kidney and other organs [50, 52], it is unlikely that the inorganic nitrite supply had detrimental effects on renal function in the present study. Clearly, the present data show that the organic nitrites are superior to inorganic nitrite in renal IRI at the doses used. Finally, the sheep used were anesthetized using isoflurane. This has previously been shown to aggravate renal vasoconstriction in endotoxemia-induced AKI [53]. However, this effect was attenuated with major fluid resuscitation and here renal blood flow reached levels comparable to the conscious state [54].

## Conclusions

Our study demonstrated that the novel NO donor PDNO significantly improved renal function during ischemia reperfusion in a large-animal experimental model. This was accompanied by an improved renal medullary perfusion indicating a direct vasodilatory effect of PDNO on the microcirculation. PDNO also increased renal oxygen consumption which in combination with disrupted perfusion could be detrimental. However, a more effective oxygen handling, as indicated by a lower ratio between consumed oxygen and reabsorbed sodium, instead suggested less renal tubular damage and replacement of pathologically reduced renal NO levels in the PDNO group. The beneficial renal effects of PDNO in combination with absent methemoglobinemia, tachyphylaxis, or severe hypotension may make PDNO a potentially interesting NO donor for treatment of certain forms of renal IRI.
